# FETP-to-system integration model: a health system resilience model in the Middle East and North Africa

**DOI:** 10.3389/fpubh.2026.1789792

**Published:** 2026-03-18

**Authors:** Engy M. El-Ghitany, Salma K. Saad, Eman A. Omran

**Affiliations:** 1 Department of Tropical Health, High Institute of Public Health, Alexandria University, Alexandria, Egypt; 2 National Committee of Epidemiology - Academy of Scientific Research & Technology, Cairo, Egypt; 3 Faculty of Medicine, Alexandria University, Alexandria, Egypt; 4 Department of Microbiology, High Institute of Public Health, Alexandria University, Alexandria, Egypt

**Keywords:** field epidemiology training programs (FETPs), health security, health system integration, health system resilience, international health regulations (IHR), Middle East and North Africa (MENA), workforce governance

## Abstract

**Background:**

Field Epidemiology Training Programs (FETPs) are a core element of applied epidemiology capacity worldwide. FETPs in the Middle East and North Africa (MENA) region, have repeatedly demonstrated their value during public health emergencies. However, their long-term impact is still doubtful. This study is an attempt to view how FETPs are positioned within national health systems in the MENA region, explore why sustainability challenges persist despite clear programmatic success, and identify potential pathways for institutionalizing FETPs.

**Methods:**

A conceptual analytical approach was applied, relying on published evaluations of FETPs, global health system, workforce governance and international health security principles.

**Results:**

The study shows that FETPs almost consistently strengthen disease surveillance and outbreak response capacity across the region. However, these gains are frequently episodic rather than institutionalized. Sustainability can be constrained by weak alignment between FETP training outputs and national workforce governance structures, limited policy and financing embedding, and the health security action is probably reactive instead of the preferable proactive preparedness. These challenges can further be intensified by the political and epidemiological situations of the MENA region.

**Conclusions:**

Sustainability challenges facing FETPs in the MENA region may stem primarily from structural misalignment rather than programmatic weakness. The proposed “FETP-to-System Integration Model” provides a potential strategic proposal for strengthening sustainability. Repositioning FETPs as long-term system investments, rather than episodic emergency response assets, is believed to be essential for enhancing health system resilience and regional health security.

## 1 Introduction

Field Epidemiology Training Programs (FETPs) have globally gained widespread acceptance over the last 40 years as a key tool for supporting epidemiological capacity and eventually strengthening public health systems including preparedness for health emergencies (1, 2). FETPs are competency-based, field-focused, learning-through-service model approaches. Their main objectives include gaining practical experiences in disease surveillance, outbreak investigation, and evidence-based decision-making in a variety of contexts (3, 4). Therefore, they are believed to promote global, regional and national health security (5). FETPs are an important part of Essential Public Health Functions (EPHFs) which are a framework that defines the core activities that public health systems must deliver to protect and improve population health. The unified list of EPHFs consists of 12 activities that can be used to operationalize public health in a country including surveillance, laboratory systems, workforce development, and emergency preparedness (6). Success of FETP reinforces it as a mechanism for strengthening essential public health functions in countries (7).

The Middle East and North Africa (MENA) region has a unique political and epidemiological profile that shapes the environment in which health systems function. It is characterized by long-term conflict, political instability, population displacement, climate vulnerability, donor dependency, recurrent public health emergencies, variable institutional capacities and a quickly changing epidemiological pattern that includes both newly emerging infectious diseases and an increasing burden of non-communicable diseases (8, 9). FETPs have been used in numerous nations in this complicated setting, and they have proven useful in times of public health emergency. In such contexts, understanding sustainability requires attention not only to technical performance, but also to governance, and health system resilience (9, 10).

Across the literature, FETPs showed widespread adoption and documented short-term successes. On the other hand, there is a growing recognition of scientific gaps in the literature regarding system-level integration, institutionalization, evaluating FETP effectiveness, sustainability, and long-term impact. Identifying and addressing these gaps is essential for optimizing FETPs role, ensuring sustainable impact, and maximizing contributions to global health security (11, 12).

FETPs should be integral in all phases of health systems: prevention, preparedness, response, and recovery. Health system recovery is defined as the rebuilding, restoration and improvement of the health system's components and core public health functions, in alignment with the principles of build back better and sustainable development. This entails rehabilitation of infrastructure, restoration of services, workforce support, resilience building, and responding to community health needs (13). During the COVID-19 pandemic, FETPs had a vital importance in emergency response. Meanwhile this pandemic underscored the necessity of repositioning FETPs as continuous epidemic intelligence asset instead of being episodic responders having a primarily response-oriented path of adaptability (14, 15).

The CDC, in their “FETP-Frontline Curriculum Guide” determined standards for FETPs regarding their duration, learning objectives, structure and timeline, mentorship, workshops and fieldwork formulations as well as modes of assessment of participants. Improper adherence to such standards might compromise the traniee's expertise, and eventually impair the integration and contribution to health system resilience (3). Recently, several publications studied FETPs, and most of them aimed at program assessment and covered a short time-span without longitudinal assessment of the long term public health impact. Al Nsour et al. (1) conducted a global scoping review that synthesized evidence from published evaluations of FETPs and confirmed their positive impact on trainee competencies, surveillance functions, and outbreak response. The review mainly examined how FETPs have been evaluated also highlighted limitations in evaluation approaches and the need for standardized metrics.

The long-term contribution of FETPs to health system resilience is believed to rely primarily on early institutional embedding. Recent analyses from the region demonstrated that although FETPs almost consistently strengthen surveillance capacity and emergency response performance, training-led capacity building alone is insufficient to ensure sustainability when governance, financing, and workforce absorption mechanisms are not addressed in parallel (16).

In order to close these gaps, this paper offers a system-oriented, region-specific analysis of FETPs in the MENA region, going beyond evaluation of the short-term results of the programs themselves. The study looks at FETPs from the perspectives of the health system, workforce governance, and health security in order to determine how to institutionalize FETPs as long-lasting elements of national health systems and to explain why sustainability issues continue despite programmatic success. In order to direct future policy and practice, the paper suggests a conceptual FETP-to-System Integration Model and how programs should change to address new health security threats.

## 2 Methods

### 2.1 Study design

In this paper, we adopted a conceptual narrative approach to structurally analyze the alignment between Field Epidemiology Training Programs (FETPs) and national health systems in the Middle East and North Africa (MENA) region. The paper focuses on studying the availability and adequacy of data on FETPs sustainability and long term effect. We used a thematic analysis across selected resources to extract recurring patterns of integration, governance, and sustainability. This approach was chosen due to the heterogeneity of published data and hardship to develop systemically quantitative metrics. In this context, we tried to address the following questions:

(i) **what** FETPs position is within national health systems;
(ii) **why** sustainability challenges persist despite strong functional performance; and
(iii) **how** FETPs can be more effectively institutionalized using established health system and health security frameworks.

### 2.2 Data sources

To develop the proposed concepts, we relied on three complementary sources of evidence (Supplementary Table S1). They were selected to balance empirical documentation, normative guidance, and regional context.

First, published evaluations and reviews of FETPs: The sources used in this context included regional and global ones. The examination focused on recording how programs contributed to surveillance, managing outbreaks, while also shaping capacity in terms of workforce. These sources included regional and global figures that provided the empirical basis for identifying recurring strengths, limitations, and patterns across diverse country contexts (1, 2, 10, 17) Secondly, the World Health Organization normative frameworks, which we used as benchmarks for assessing system alignment. These included the WHO health system building blocks framework (18), the Global Strategy on Human Resources for Health (Workforce 2030) (5), and the International Health Regulations (2005) (19). Together, these frameworks define internationally recognized standards for governance, workforce integration, and health security operation.

Thirdly, evidence from the MENA region was incorporated to ensure that global frameworks were interpreted in view of regional realities. This included case studies that were considered empirical studies from countries in the regional context to ground the analysis in concrete country experiences. These reported documented experiences from Jordan, Egypt, and Yemen (Table 1) which illustrate both successful institutionalization pathways and persistent fragility (20–23). Additionally, the WHO Regional Office for the Eastern Mediterranean (EMRO) reports on health systems in fragile and conflict-affected settings, emergency preparedness, and pandemic response were included in our analyses (24–27).

**Table 1:** Case summaries of FETP successes in Yemen (20), Egypt (22), and Jordan (21).

**Country**	**Examples of medical conditions/outbreaks tackled during the FETP**	**Programmatic successes**	**Limitations/Fragility**
Yemen	COVID-19, cholera, measles, acute flaccid paralysis, diphtheria	60 to 80% of graduates conducted outbreak investigations, surveillance analysis/evaluation, managed surveillance systems/projects, engaged in public health communication (reports/presentation), and used basic statistical methods.	-primarily funded by donors; thus, was not sustainable. -low graduate retention. -limited training in policy development and management.
Egypt	Avian influenza- foodborne illness outbreaks in three governorates and suspected viral haemorrhagic fever cases. Furthermore, RRT participated in multiple mass gathering events in Egypt such as United Nations Climate Change Conference (COP27).	Significant improvements in monitoring and evaluation and post-deployment capacity levels. For operational levels, statistically significant increases were observed across most areas, with the greatest improvements in post-deployment, activation and pre-deployment and deployment.	
Jordan	- foodborne illness. - Middle East respiratory syndrome coronavirus (MERS-CoV). - collecting and reporting data regarding Syrian refugee care at Ministry of Health facilities. - J-FETP has led the implementation of 3 behavioral risk factors surveillance system surveys.	Improved surveillance systems, including revising the mortality surveillance policy, implementing the use of electronic data reporting, investigating outbreaks at national and regional levels, contributing to non-communicable disease research and surveillance, and responding to regional emergencies and disasters.	There remains a shortage of skilled epidemiologists in Jordan. Political unrest and humanitarian crises in the region add complexity to the public health needs in Jordan.

### 2.3 Analytical framework

The aim of the analysis was to identify structural gaps, recurring patterns, and the degree of alignment between FETPs and health system functions. Therefore, evidence from these sources was interpreted using three **complementary analytical lenses**, applied iteratively across the literature.

The first lens, **health system integration**, examined the extent to which FETPs are embedded within national health systems. This included their linkage to governance, financing mechanisms, routine surveillance functions, and health information system oversight.

The second lens, workforce governance, assessed human resources strategies in national health systems in terms of position definitions, career pathways, accreditation, and retention in relation to competencies produced through FETP training.

The third lens, health security positioning, explored how FETPs are situated within preparedness, response, and epidemic intelligence architectures. Special attention was given to whether they function primarily as surge response or as continuous contributors to health security systems. Particular attention was given to the role of FETPs across the full emergency cycle, including preparedness, response, and recovery. Recovery was operationalized as activities contributing to restoration and strengthening of routine surveillance systems, after-action reviews, institutional learning, system reform, and continuity of essential public health functions following public health emergencies.

Importantly, the analysis considered interactions between lenses, for example, how misalignment in workforce governance can constrain functional integration or undermine sustainability, rather than treating each dimension in silos.

### 2.4 Model derivation

The **FETP-to-System Integration Model** was developed through a structured synthesis of the analytical findings, rather than through the creation of a new theoretical framework. As evidence was examined across the three analytical lenses, we concluded four interdependent domains which repeatedly emerged as critical determinants of sustainability and system-level impact.

The four pillars; institutional anchoring, workforce alignment, functional integration, and adaptive scope, structured the proposed model. Each of them corresponds to a documented gap and informed by WHO frameworks. For example, “institutional anchoring” addresses the frequent project-based style of FETPs use, while “adaptive scope” responds to the evolving risk in MENA. Together, they capture the structural conditions under which FETPs are most likely to transition from effective training programs to durable components of resilient health systems.

### 2.5 Ethics considerations

As this study is based exclusively on secondary analysis of publicly available literature and policy documents, ethical approval was not required.

## 3 Results and discussion

This analysis applied a systems-oriented perspective to FETPs in the MENA region to explain why clear programmatic success has not consistently resulted in sustained institutionalization. In this analysis, we shifted the focus from evaluating training outcomes to the governance, workforce, and health security environments in which FETPs operate.

A consistent pattern was shown across the MENA region when we applied the health system integration, workforce governance, and health security perspectives. FETPs demonstrate clear programmatic and functional success relevant to graduates capacities which was usually reflected on strengthening surveillance and outbreak response (1). Unfortunately, this success has not been translated into sustained institutionalization within health systems.

Our analysis indicates that this gap can be explained by structural misalignment between FETPs and health systems architecture and not by limitations in training quality or quantity. This observation is more evident when health systems operate in fragile and emergency-prone contexts (16). Evidence from Egypt further illustrates that improvements in operational performance may not be sustained when administrative, policy, and financing dimensions are not addressed early in program design (22). This justifies the inclusion of institutional anchoring and functional integration pillars of the proposed model.

### 3.1 Health systems patterns in relation to FETPs

**Figure 1:**
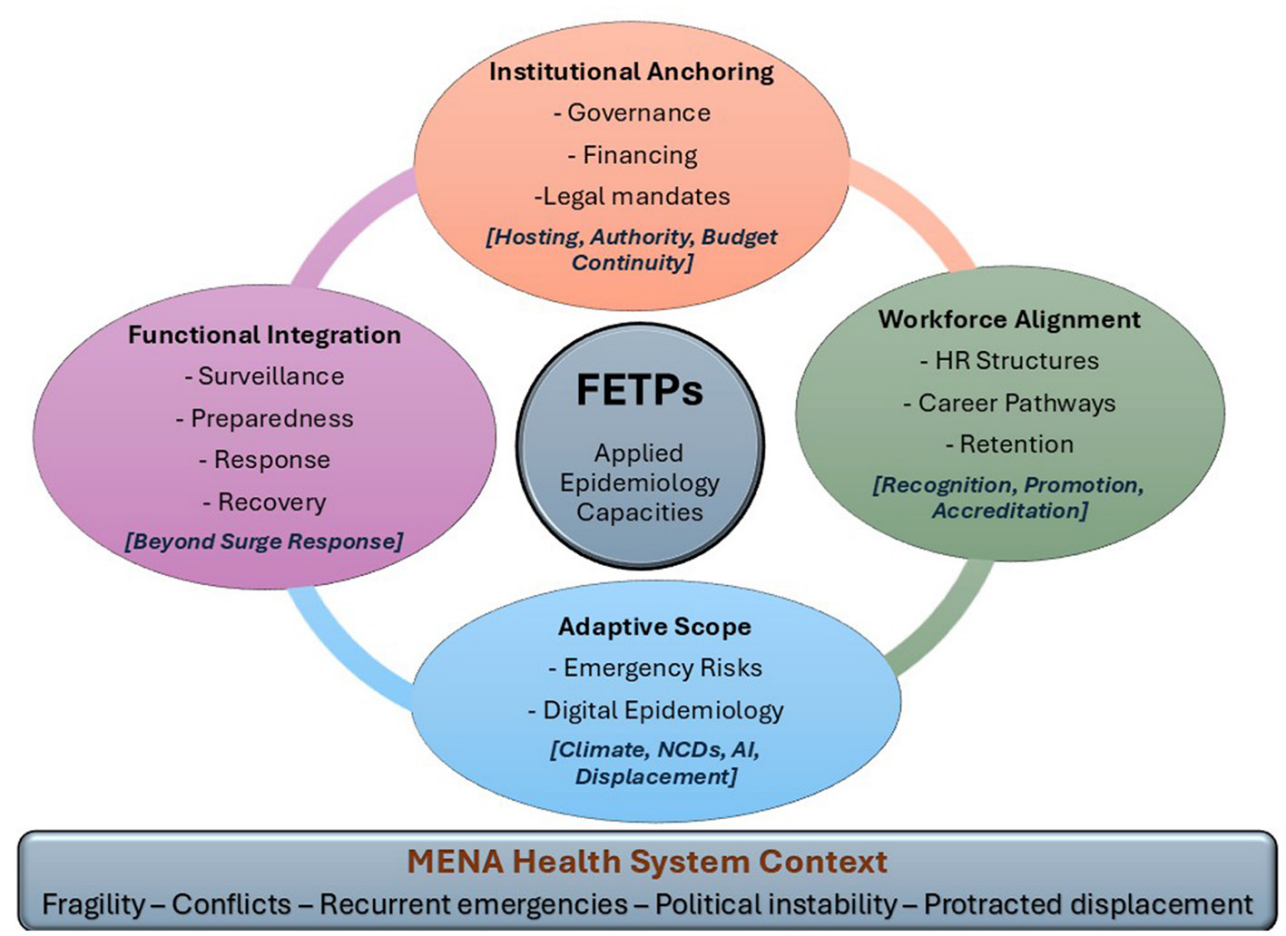
FETP-to-system integration diagnostic & operational model for health system resilience and health security in the Middle East and North Africa. This figure illustrates the FETP-to-System Integration Model. It depicts the study conceptualization of how Field Epidemiology Training Programs (FETPs) can be positioned as sustainable components of national health systems and health security architecture in the MENA region. The model comprises four interdependent pillars; institutional anchoring, workforce alignment, functional integration, and adaptive scope. The figure explains briefly the targeted health system elements in each pillar and how to tackle for shaping the sustainability and system-level impact of FETPs. The below contextual layer reflects the regional realities including unstable political context of the MENA region with subsequent evolving health risks. The model functions as both a diagnostic and operational framework for identifying misalignment and guiding system-level reforms.

The following patterns were extracted in this analysis and constituted the foundation for development of the four interdependent pillars of the proposed FETP-to-System Integration Model (Figure 1) in this study.

#### 3.1.1 Positioning of FETPs within health systems: functional strength vs. weak system embedding

FETPs consistently contribute to core public health functions, including disease surveillance, outbreak investigation, and emergency response through improvements in qualities relevant to data analysis, reporting and communication (1, 2). During those events, FETP trainees and graduates are actively mobilized.

When viewing through a health system lens, FETPs are positioned primarily as functional assets that work as episodic system enhancers whose influence tends to peak during emergencies. They usually do not constitute institutional components and are insufficiently embedded in routine system performance (11).

#### 3.1.2 Workforce governance: training gains without system absorption

As mentioned earlier, evaluations consistently document strong gains among FETP graduates. Meanwhile, a gap persists between the competencies generated through FETPs and the ability of health systems to absorb, retain, and deploy them (28, 29).

In some settings, access to FETPs is uneven. FETP specialists sometimes favor specific cities, specialties, or institutions. More broadly, applied epidemiology is often not recognized as a distinct professional track. FETP completion does not consistently influence recruitment, promotion, or remuneration pathway in many MENA countries. This may explain training–employment–retention paradox (28, 30, 31).

Empirical evidence shows that this gap can be addressed as was demonstrated in Jordan's experience. Formal recognition of FETP training within national workforce governance systems enabled graduates to move into leadership and decision-making roles (21). Conversely, the lack of workforce alignment increases attrition and underutilization of skills. This is usually shown in emergency-prone contexts where temporary funding, project-based posts, and parallel systems predominate. These findings are consistent with WHO workforce governance priorities. They entail that sustainability, beside training outputs, depends on integrating competencies into formal workforce structures, career pathways, and retention mechanisms (5, 19).

Accordingly, workforce alignment constitutes a central pillar of the proposed model, which links individual capacity development to system-level resilience.

#### 3.1.3 FETPs within health security architecture: reactive vs. proactive integration

Viewed through a health security lens, many health systems in the MENA region are response-based and emergency-driven in nature, and so is the utilization of FETPs as described earlier (1). While this role is essential, an exclusively reactive rather than proactive system limits the FETPs strategic potential as a sustainability asset.

Despite that emergency preparedness, risk assessment, simulation exercises, and post-event learning are crucial functions to resilient health systems, yet FETPs showed effectiveness in these aspects, their utilization remains limited (16). The MENA region has a variety of evolving risks, such as climate-dependent health threats, non-communicable diseases, humanitarian crises, and digital transformation of surveillance systems. These context-specific realities mandate the adoption of an integrated adaptive scope pillar in the proposed model. This pillar is also supported by WHO guidance on emergency risk management, epidemic intelligence, and digital health, and emphasizes strategic adaptation (32).

The functional integration pillar of our model emphasizes embedding FETPs across the full preparedness–response-recovery spectrum. This is consistent with international health security frameworks and would strengthen their role as permanent contributors to a sustainable resilient system, not solely as surge capacity (16).

Recent experience from Egypt provides empirical validation of this approach. The authors showed that emergency epidemiology capacity is most effective and sustainable when training initiatives are accompanied by governance and integration reform (22). Similarly, the documented involvement of Jordan FETP graduates in routine surveillance reform, outbreak response, and refugee health monitoring demonstrates the feasibility of sustained functional integration in MENA settings (21).

#### 3.1.4 Adaptive scope: aligning FETPs with evolving regional risks and opportunities

The adaptive scope is inevitable in a world full of evolving events and significant shifts. Traditional outbreak-focused training remains essential. However, it is no longer sufficient on its own to address intersecting risks (5, 16).

The adaptive scope pillar implies strategic resilient alignment with national risk profiles and emerging system needs. WHO guidance on emergency risk management, epidemic intelligence, and digital health supports a shift toward strengthened analytical and resilient capabilities (25, 26). Importantly, evidence on field epidemiology pedagogy confirms that learning-by-doing models remain effective across contexts. Future challenges would relate less to training design than to system readiness to deploy evolving competencies (4, 22).

### 3.2 Toward the FETP-to-system model integration: synthesizing the evidence

After synthesizing findings across the three analytical perspectives, it was clarified why FETPs continue to face sustainability challenges despite clear evidence of programmatic success. The probable answer can be summarized as follows;

- Effective FETPs capacities are not necessarily matched by structural institutional anchoring within health systems;
- Workforce governance mechanisms do not consistently recognize or retain applied epidemiology expertise; and
- Emergency-driven deployment limits the strategic use of FETPs within broader health security systems (16).
  These challenges might be amplified, given the region's fragility context.

Countries in the region showed different experiences based on the adopted health system. Experience from Yemen shows high functional contributions during conflict. However, weak institutionalization undermines sustainability once external support diminishes (20, 23). In contrast, evidence from the Eastern Mediterranean Region demonstrates that this trajectory is not inevitable. The experience of the Jordan FETPs illustrates how early and deliberate institutional anchoring within health system can enable long-term sustainability and national ownership (21). When we take these contrasting experiences together, it helps us to conceptualize that institutional anchoring is not an administrative detail, but a foundational condition for sustainability.

#### 3.2.1 Introducing the model

The FETP-to-System Integration Model is a diagnostic and interventional tool. It is designed to address the persistent gap between the program success and institutional sustainability. The four interdependent pillars of the proposed FETP-to-System Integration Model namely; institutional anchoring, workforce alignment, functional integration, and adaptive scope, are illustrated in Figure 1. Table 2 further operationalizes each pillar by showing system-level focus, misalignment and evidence.

**Table 2:** Operational interpretation and evidence of the FETP-to-system integration model.

**Model pillar**	**System-level focus**	**Manifestation of misalignment**	**Illustrative evidence**
Institutional anchoring	Governance, financing, legal mandate.	FETPs hosted as projects; absence of protected budget lines; unclear administrative authority.	Externally supported or donor-dependent programs; contrast between sustained institutionalization in Jordan and fragility in Yemen (20, 21, 23, 33).
Workforce alignment	HR structures, career pathways, retention.	FETP completion not linked to job classification, promotion, or remuneration; reliance on temporary or project-based posts.	Strong competency gains; weak workforce absorption; national accreditation and career recognition in Jordan (9, 21).
Functional integration	Surveillance, preparedness, response.	FETPs mobilized primarily during outbreaks; limited involvement in routine surveillance governance and preparedness planning.	Response-driven deployment; pandemic lessons highlighting gaps in continuous epidemic intelligence (25).
Adaptive scope	Dealing with evolving risks	Growing demands related to climate, NCDs, displacement, and digital surveillance.	WHO guidance on emergency risk management and epidemic intelligence; regional evidence of expanding epidemiological complexity (16, 26).

#### 3.2.2 Pillar 1: institutional anchoring

It refers to the formal embedding of FETPs within national governance and financing structures. This pillar addresses the common vulnerability of FETPs as externally funded projects by advocating for clear legal mandates and protected budget lines. This ensures transition from temporary initiative to built-in process.

#### 3.2.3 Pillar 2: workforce alignment

It focuses on integrating FETPs graduates into HR structure of the health system. This involves establishing recognized job classifications, career pathways and incentives structure. This pillar resolves the training-employment paradox and transforms FETPs into a rewardable professional track.

#### 3.2.4 Pillar 3: functional integration

Functional Integration ensures that FETPs gains are embedded in the daily operations of the health system. It emphasizes routine involvement in surveillance, preparedness, response, and recovery. This shifts the utilization of FETPs from episodic to continuous response.

#### 3.2.5 Pillar 4: adaptive scope

Adaptive Scope is the model's forward-looking component. It ensures the FETPs remain relevant and keep up with future threats and advancement (or coping evolving updates). These updates may include climate-related health issues, displacement, NCDs, modern technologies, or any other shifts.

### 3.3 Policy and practice implications

The four pillars of the FETP-to-System Integration Model provide, from the authors' perspective, a coherent explanation for why sustainability challenges persist despite the clear program success (1). The model is not intended as a prescriptive blueprint, but as an analytical and planning tool that can support policymakers and program leaders in diagnosing misalignment, prioritizing reforms, and sequencing investments in ways consistent with health system and workforce governance principles (5, 19, 26).

Policymakers and program leaders can use the model to diagnose misalignment and prioritize intervention(s). To operationalize this model, actionable steps should be taken for each pillar, such as drafting legal mandates for institutional anchoring and reforming HR regulations for workforce alignment. For donors, this implies shifting investment from standalone training to system-integration actions.

The model shifts attention from expanding training volume to strengthening the system conditions. This would strengthen resilience, and long-term impact. In the MENA region, where health systems often operate under conditions of uncertainty, this structural shift is critical for translating investments in applied epidemiology into sustained and resilient health security gains (16).

### 3.4 Limitation

This study was based on conceptual and not systematic analysis which may reflect publication bias or underrepresentation of certain areas.

The proposed model requires pilot implementations and empirical validation.

The analysis did not provide stepwise roadmap scenarios to tackle each misalignment.
