# Supplementary material for: FETP-to-system integration model: a health system resilience model in the Middle East and North Africa
Source: Front Public Health. 2026 Mar 18;14:1789792. doi: 10.3389/fpubh.2026.1789792 (PMC13066136; doi:10.3389/fpubh.2026.1789792)
Supplement: Supplementary file 1 [file Table_1.docx]

**Supplementary Table S1: Categories of documents reviewed and analytical purpose**

| **Document category** | **Source type** | **Reference numbers of some examples** | **Analytical purpose** |
| --- | --- | --- | --- |
| FETP program evaluations | Peer-reviewed articles | 1, 2 , 10, 11, 12, 14, 15,17, 29, 30, 31, 33 | Identify documented programmatic performance, strengths, and reported sustainability challenges |
| Health system frameworks | normative guidance | 3, 6, 7 , 8 , 9, 13, 18, 28, 32 | Benchmark institutional anchoring, governance, and financing alignment |
| Workforce governance frameworks | global strategies | 4, 5 | Assess alignment between FETP outputs and national HRH structures |
| Health security frameworks | International agreements & guidance | 16,19 | Examine positioning of FETPs across preparedness, response, and recovery functions |
| Regional health system analyses | WHO- EMRO reports | 24- 27 | Integrate MENA-specific political, institutional, and epidemiological context |
| Country-specific empirical studies | Peer-reviewed regional studies | 20,21,22,23 | Ground the model in real-world regional experience and variability |
